# Emerging Threats for Human Health in Poland: Pathogenic Isolates from Drug Resistant* Acanthamoeba* Keratitis Monitored in terms of Their* In Vitro* Dynamics and Temperature Adaptability

**DOI:** 10.1155/2015/231285

**Published:** 2015-11-23

**Authors:** Lidia Chomicz, David Bruce Conn, Marcin Padzik, Jacek P. Szaflik, Julia Walochnik, Paweł J. Zawadzki, Witold Pawłowski, Monika Dybicz

**Affiliations:** ^1^Department of Medical Biology, Medical University of Warsaw, 73 Nowogrodzka Street, 02-018 Warsaw, Poland; ^2^Department of Invertebrate Zoology, Museum of Comparative Zoology, Harvard University, Cambridge, MA 02138, USA; ^3^One Health Center, Berry College, School of Mathematical and Natural Sciences, Mount Berry, GA 30149-5036, USA; ^4^Department of Ophthalmology, SPKSO Ophthalmic Hospital, Medical University of Warsaw, 13 Sierakowskiego Street, 03-709 Warsaw, Poland; ^5^Center for Pathophysiology, Infectiology and Immunology, Institute of Specific Prophylaxis and Tropical Medicine, Medical University of Vienna, Kinderspitalgasse 15, 1090 Vienna, Austria; ^6^Clinic of Cranio-Maxillo-Facial and Oral Surgery and Implantology, 4 Lindleya Street, 02-005 Warsaw, Poland; ^7^Department of Disaster Medicine, Warsaw Medical University, 81 Żwirki and Wigury Street, 02-091 Warsaw, Poland; ^8^Chair and Department of General Biology and Parasitology, Medical University of Warsaw, 5 Chałubinskiego Street, 02-004 Warsaw, Poland

## Abstract

Amphizoic amoebae generate a serious human health threat due to their pathogenic potential as facultative parasites, causative agents of vision-threatening* Acanthamoeba* keratitis (AK). Recently, AK incidences have been reported with increasing frequency worldwide, particularly in contact lens wearers. In our study, severe cases of AK in Poland and respective pathogenic isolates were assessed at clinical, morphological, and molecular levels. Misdiagnoses and the unsuccessful treatment in other ophthalmic units delayed suitable therapy, and resistance to applied chemicals resulted in severe courses and treatment difficulties. Molecular assessment indicated that all sequenced pathogenic corneal isolates deriving from Polish patients with AK examined by us showed 98–100% homology with* Acanthamoeba* genotype T4, the most prevalent genotype in this human ocular infection worldwide.* In vitro* assays revealed that the pathogenic strains are able to grow at elevated temperature and have a wide adaptive capability. This study is our subsequent* in vitro* investigation on pathogenic* Acanthamoeba* strains of AK originating from Polish patients. Further investigations designed to foster a better understanding of the factors leading to an increase of AK observed in the past years in Poland may help to prevent or at least better cope with future cases.

## 1. Introduction

Amoebae belonging to the genus* Acanthamoeba* are ubiquitous and widely distributed in natural and man-made environments worldwide.* Acanthamoeba* spp. are free-living organisms existing as vegetative mononuclear trophozoites with characteristic acanthopodia and as double-walled dormant cysts, developing after the growth phase as well as under harsh conditions. The protists occur in sea, fresh, tap-water, and drinking water systems and in swimming pools, air conditioning systems, and humidifiers; they also occur in dust and soil, on fruits and vegetables, and in animal bodies. They have been recognized in the hospital environment as contaminants of surgical instruments and dental irrigation units, as well as in various human cavities and tissues, and on skin surfaces, oral cavities, paranasal sinuses, lungs, and brain [1–4]; trophozoites and cysts of* Acanthamoeba* also have been found by us among the microbiota of periodontal biofilms, accompanying infections with* Entamoeba gingivalis* in patients with systemic diseases [5].

The free-living amoebae complete their life cycles in different external environments, without entering humans or animals, and feed on microorganisms and small organic particles. However, in some circumstances, they are able to enter human bodies from different sources, colonize some organs, multiply within them, and thus exist as opportunistic parasites causing pathogenic effects. Epidemiological, serological, biochemical, and molecular investigations have shown that people may be exposed to pathogenic as well as nonpathogenic* Acanthamoeba* strains [3, 6]. It seems that the amoebae come into the human body relatively frequently, without pathogenic consequences, as indicated by 50–100% of the healthy population having specific antibodies [7–9]. However,* Acanthamoeba* spp. may be causative agents of the rare but usually fatal granulomatous amoebic encephalitis, developing in immunocompromised individuals as an opportunistic infection [4], and of the vision-threatening* Acanthamoeba* keratitis (AK) that occurs mainly in immune-competent persons. AK was first recognized in 1973 in a Texas rancher [10]. The eye disease symptoms include redness, photophobia, excessive tearing, severe eye pain, and significant deterioration of the visual acuity; without adequate therapy the amoebic infections may lead to blindness [3, 10–17].

The clinical symptoms of AK are nonspecific, similar to those observed in the course of other eye diseases, thus misdiagnosed as viral, fungal, or bacterial keratitis; a mixed keratitis caused by concomitant bacterial, viral, fungal, and* Acanthamoeba* infections is also known. This is why the diagnosis based on clinical symptoms alone is not sufficient to indicate the causative agent of human keratitis. The proper diagnosis needs laboratory identification of the specific pathogen for confirmation. Corneal scrapings are optimal materials for AK diagnosis. The microscopic visualization of amoebae in slides prepared directly from corneal scraping and by* in vitro* cultivation of the amoebic isolates deriving from these samples may be helpful also to verify previous misdiagnoses [3, 17, 18].

For years,* Acanthamoeba* isolates/strains were classified based on morphological criteria, mainly cyst size and structure: three morphological groups and 18* Acanthamoeba* species were determined [3, 19, 20]. In the recent past, with the development of molecular systematics, PCR techniques and sequence analysis of the 18S rRNA gene have been used for diagnostics and for characterization of clinical and environmental* Acanthamoeba* isolates [4, 21–25].

At present, 19 genotypes are distinguished [17].

The treatment of AK is difficult, and often a resistance to pharmacotherapy develops, among other factors, due to an improper diagnosis leading to delayed suitable therapy. Moreover, the amoeba cysts are highly resistant to chemicals: disinfectants and antimicrobial and antiparasitic drugs; antiamoebic drugs are often efficient in high concentrations, which, however, are toxic for human cells [3, 4, 26–30]. Thus, despite therapeutic advances, the treatment of the keratitis caused by pathogenic* Acanthamoeba* strains continues to be difficult and is often unsuccessful.

In our earlier studies of 2011–2014 [16, 31], several pathogenic* Acanthamoeba* strains acquired from serious AK cases, variable in corneal symptom intensity and in the response to the instituted therapy, were assessed. The corneal strains have been classified as morphological group II and, in genotype identification carried out by us with PCR based on sequence analysis of the 18S rRNA, determined as T4 genotypes.* In vitro* viabilities of particular strains were monitored and compared. Results of the monitoring we analyzed in regard to the survival time of amoebae, AK course as well as therapeutic management difficulties/efficacies [16]. It has been revealed in our preliminary studies that monitoring of* in vitro* viability of pathogenic* Acanthamoeba* strains isolated from the infected eyes may be a useful tool for therapeutic prognosis.

A temperature tolerance of the amoebae, particularly growth/multiplication at high temperature, is often studied and reported, because it is considered as an indirect marker of potential pathogenicity of* Acanthamoeba* strains [3, 17, 32, 33]. However, apart from our preliminary investigations, no studies were undertaken with diagnosed pathogenic* Acanthamoeba* strains originating of AK in terms of their* in vitro* viability in changed temperature conditions.

In the present study, subsequent pathogenic* Acanthamoeba* isolates acquired by us from human* Acanthamoeba* keratitis cases unsuccessfully treated with antibacterial and antifungal medications and poorly responding to topical antiamoebic pharmacotherapy, assessed at clinical, morphological, and molecular levels, were* in vitro* monitored. The corneal isolates diagnosed as pathogenic* Acanthamoeba* strains were examined and compared to one another as well as to the environmental* Acanthamoeba castellanii* Neff strain in terms of their* in vitro* temperature sensibility/tolerance. Moreover, dynamics of these amoebic populations, cultivated parallel* in vitro*, their density, and morphophysiological status of particular developmental stages were assessed.

## 2. Materials and Methods

### 2.1. *Acanthamoeba* Pathogenic Corneal Strains: Isolates and Cultures

The material deriving from twenty-two patients who reported to our hospital in 2010–2014 at different times after first symptoms of keratitis appeared and who were under suspicion of* Acanthamoeba* infection was analyzed. The patients complained of photophobia, pain, excessive tearing, and deterioration of visual acuity. In the clinical diagnosis, noninvasive methods of slit-lamp and* in vivo* confocal microscopy were used. During the laboratory microbiological and parasitological diagnosis, direct microscopic examinations of corneal scraping material and* in vitro* cultures derived from those scrapings were performed to determine etiological agents of the eye deteriorations. Although* Acanthamoeba* infections were confirmed for all cases, the isolates marked as I-1 up to I-22 showed a high degree of variation.

In ten cases that were properly diagnosed early, 3 to 15 days after first AK symptoms appeared, and thus received early suitable treatment, an improvement was observed relatively quickly. As the isolates showed* in vitro* weak dynamics, trophozoites were dead in the culture medium after 8–10 days, no transformation into cysts was observed, and thus no material from these ten strains was kept for further* in vitro* investigations.

Another two isolates have originated from incidences in which AK has been diagnosed and pathogenic amoebae were isolated; however after moderate improvement, the two patients have not continued therapy and no information on treatment efficacy was available.

For these reasons, finally, ten remaining isolates acquired from the corneal material have been included in this analysis.

The patients, three men, all contact lens wearers, and seven women, six contact lens wearers, one of whom bathed in swimming pool, and one non-contact-lens wearer with a history of swimming in a lake, all 26–42 years old, had different intensities of pathogenic changes in their eyes. All of them had previously been unsuccessfully treated in other ophthalmic units with antifungal and antibacterial pharmaceutics; thus proper diagnosis was delayed ranging from 25 to 45 days after first symptoms appeared.

The initial identification of causative agents was achieved by* in vivo* confocal microscopy. The final diagnosis was made/confirmed by corneal scrapings examinations in the light microscope, first directly and next with enrichment during* in vitro* cultivation; the isolates were assessed at cytological and molecular levels.

The isolates originating from corneal samples of the AK patients, initially examined in wet-mount slides to visualize cysts or/and trophozoites of amoebae, were cultured under bacteria-free conditions for one to three years in sterile 15 mL tubes containing BSC culture medium [34] enriched with 10% calf serum, incubated at 27°C, and subcultured twice each month.

Additionally, environmental* A. castellanii* Neff strain, after years with serial passages in the same growth medium in the Laboratory of the Department of Medical Biology, Medical University of Warsaw, Poland, was used in this study.

### 2.2. Genotyping of* Acanthamoeba* Strains

All samples/isolates were also examined by PCR techniques for specific detection of* Acanthamoeba* DNA and to determine genotypes of the particular strains. Extraction of DNA from the samples was performed using commercial Sherlock AX Kit (A&A Biotechnology, Gdynia, Poland). Extraction of DNA from cultured* in vitro* isolates was performed using commercial Genomic Mini Kit (A&A Biotechnology) for routine genomic DNA extraction, according to the manufacturer's instructions. Then, DNA was stored at −20°C. An* Acanthamoeba-*specific PCR following the protocol established by Schroeder et al. [21] amplifying a fragment of the 18S rRNA gene with the primers JDP1 (5′GGCCCAGATCGTTTACCGTGAA3′) and JDP2 (5′TCTCACAAGCTGCTAGGGAGTCA3′) was applied. PCR products were analyzed using GelDoc-IT Imaging Systems (UVP, USA) after gel electrophoresis on agarose gel (Sigma, St. Louis, Missouri) stained with Midori Green DNA stain (Nippon Genetics Europe GmbH, Germany). Cycle sequencing was performed and sequences obtained were compared with data available in GenBank using GeneStudio Pro Software (GeneStudio, Inc., Suwanee, Georgia).

### 2.3. *In Vitro* Growth of* Acanthamoeba* Isolates at Different Temperatures

The population dynamics of the corneal and environmental* Acanthamoeba* isolates cultured* in vitro* in the aforementioned growth medium under bacteria-free conditions at 27°C was systematically monitored in terms of developmental stage status by phase-contrast light microscopy.

For temperature assays, on the second day following subculturing, all cultures were shaken intensively and one mL samples of strains were transferred to 1.5 mL Eppendorf tubes containing culture medium. Next, the samples of the respective cultured strains were exposed to either 20°C, 37°C, or 42°C during 3–7 days following regular subculturing.

*In vitro* viability and dynamics of each particular strain population were then assessed and compared.

The morphophysiological changes and overall numbers of the amoebae as well as proportion of trophozoites and cysts were microscopically determined in the exponential and stationary growth phases. During exposure to changed temperature, cultures were vigorously shaken and 10 *μ*L samples were successively taken for assessment of each isolate. The** c**hanges in overall number of amoebae and number of trophozoites and cysts were counted with the aid of a Burker hemocytometer. The ability of amoebae to multiply* in vitro* was examined; the ranges of four counts calculated for 1 mL of culture medium were compared for particular strains and assays. Results of the investigations were analyzed statistically (ANOVA, Student-Newman-Keuls method, *p* < 0.05).

## 3. Results

**Figure 1:**
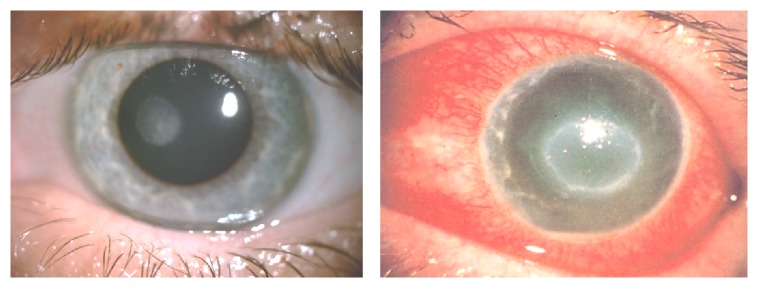
Hyperreflective tissue in corneal ulceration of severe* Acanthamoeba* keratitis cases; slit-lamp photographs.

The material assessed in our study was acquired from ten patients with symptoms of* Acanthamoeba* keratitis including redness, photophobia, severe eye pain, excessive tearing, and lid edema, as well as significant deterioration of visual acuity. Active epithelial inflammations, corneal ulcers, and characteristic ringlike stromal infiltration were detected by slit-lamp in the affected eyes (Figure 1).

Keratitis symptoms intensified in different degrees as the disease progressed.

### 3.1. Effects of Differential Diagnosis

**Figure 2:**
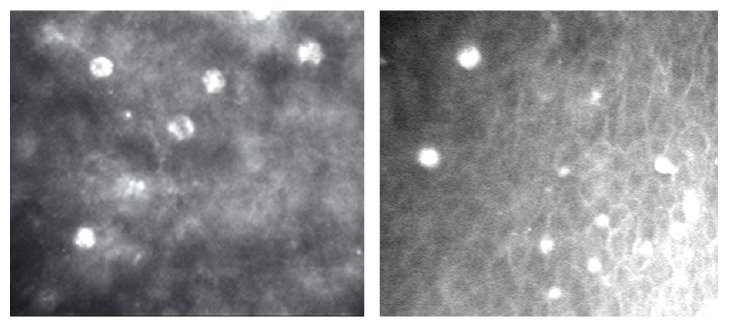
Hyperreflective objects, presumable* Acanthamoeba* cysts;* in vivo* confocal microscopy.

AK was finally confirmed in all ten cases; however, several patients experienced significant delayed proper diagnosis. In the five cases in which patients reported late to their physicians and AK diagnosis was performed more than four weeks after the first keratitis symptoms appeared, hyperreflective objects identified presumably as* Acanthamoeba* cysts by* in vivo* confocal microscopy were revealed (Figure 2).

At the same time, in 3 of five cases, amoebic, bacterial, and fungal coinfections (*P. aeruginosa, E. faecalis*, and* Candida* sp.) were revealed in the microbiological diagnosis (Table 1).

In five remaining cases, in which the duration of symptoms prior to proper diagnosis was somewhat shorter, no cysts were visualized by* in vivo* confocal microscopy.

Finally, in all samples* Acanthamoeba* infection was confirmed by laboratory methods.

In parasitological microscopic examinations,* Acanthamoeba* cysts and trophozoites were found in different materials: some of them were detected immediately in wet-mount slides prepared from corneal scrapings, whereas others were detected after 2–7 days of* in vitro* cultivation of material deriving of these isolates (Table 1).

The results of molecular examinations of the isolates and a comparison of the obtained sequences with those available in GenBank revealed that all sequenced isolates showed 98–100% homology with isolates belonging to the T4 genotype. However, there were differences between particular strains. Respective highest sequence identities were found with strains originating from both environment sources and human ocular infections and from various countries of Europe, Asia, and America.

### 3.2. Treatment Difficulties

Material included in this analysis originated from AK cases that were previously unsuccessfully treated because of improper diagnosis.

Moderate or severe course of the eye disease needed prolonged therapeutic management. Eyes affected were treated with a topically applied agent, among others, a combination of chlorhexidine (0.02%) and polyhexamethylene biguanide (PHMB, 0.02%), propamidine isethionate (0.1%), and antibiotic neomycin; however the treatment was without clear clinical improvement. Moderate response or a resistance to applied chemicals was observed during the long-term pharmacotherapy; in several cases the treatment difficulties resulted in the necessity for surgical management (corneal X-linking, penetrating keratoplasty).

### 3.3. Monitoring of* In Vitro* Dynamics of Pathogenic Corneal Isolates and Their Temperature Adaptability

Successive monitoring of the clinical isolates and comparison to the environmental* A. castellanii* Neff strain cultivated* in vitro* allowed evaluation of morphophysiological features, their associated developmental stages, and changes in their population dynamics.

**Figure 3:**
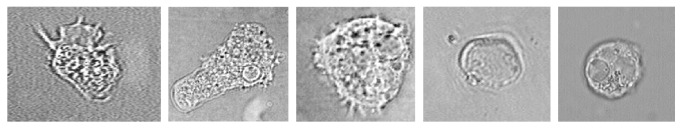
*Acanthamoeba* T4 strains, trophozoites and cysts detected in the* in vitro* cultured corneal scraping; light microscope; unstained preparations.

The living trophozoites with pseudopodia and characteristic protrusions, acanthopodia, were 12–38 *μ*m in diameter, with a nucleus and prominent centrally placed nucleolus. Cysts, 8–24 *μ*m in diameter, with their two cyst walls exhibited a wrinkled ectocyst and a polygonal or round-to-ovoid endocyst (Figure 3). The amoebae detected were identified and classified as belonging to* Acanthamoeba castellanii* morphological group II.

The comparative evaluation of monitored strains that at the beginning were cultivated* in vitro* at 27°C showed that numbers of live amoebae were low in the early adaptive phase and successively increased in the exponential growth phase while the amoebae multiplied and increased population density of particular* Acanthamoeba* strains.

The exposure of parallel cultures of strains to 20°C and 37°C from the 4th day following the subculturing caused clear changes in population dynamics, expressed in the overall number of amoeba cells.

Statistically significant differences were observed between pathogenic strains and the environmental* A. castellanii* Neff strain in terms of number of viable amoebae and thus in population density during the stationary growth phase.

Although numerous living trophozoites were detected in all cultures at 20°C, there was a significantly lower population density of pathogenic isolates cultured* in vitro* than the density of the Neff strain cultures (Table 2).

Contrary to this, the numbers of viable trophozoites in clinical isolates cultured at 37°C (this is near eye and general human body temperature, about 35°C to 37°C) were significantly higher in comparison to that found in the environmental strain. At this temperature, during the stationary growth phase, the highest numbers of amoebae were determined for I-19 pathogenic strain cultured* in vitro*, in range of four counts 44.0–102.2 × 10^2^, that were in comparison to* A. castellanii* Neff strain that were 3.3–7.8 × 10^2^ at the same temperature.

Comparative assessment of isolates exposed to 42°C showed differences between several strains. The pathogenic isolates indicated lower population activity than at 37°C, expressed in somewhat decreasing amoebic numbers.

However, in general, high amoebic population density was observed in all examined pathogenic strains during successive days of exposure to 42°C, while the number of amoebae significantly decreased in cultures of the environmental Neff strain.

Comparative data of the environmental and clinical* Acanthamoeba* strains deriving from severe cases of AK cultivated* in vitro* at 20°C, 27°C, 37°C, and 42°C are presented in Table 2.

## 4. Discussion

Recently,* Acanthamoeba* strains generate a serious human health threat due to their pathogenic potential as facultative parasites. In addition, the amoebae may also act as vehicles/sources/reservoirs of other organisms pathogenic for humans: fungal, protozoan, viral, and bacterial microorganisms which can survive and even multiply within the amoeba cells. For these reasons, epidemiological aspects are included in a majority of studies on severe vision-threatening* Acanthamoeba* keratitis. Investigations on the distribution of the virulent amoebic strains in different soil, air, and water environments have been conducted worldwide, also with the aid of molecular techniques. Potentially pathogenic strains are detected in environmental samples and reported worldwide [18, 25, 35–42].

In Poland, free-living amoebae have been isolated from waters in the vicinity of Poznań [43]. Successively, in further environmental studies on* Acanthamoeba*, potentially pathogenic strains have been detected in Lake Żarnowieckie, the Piaśnica River, and a canal used as a recreational resort in northern Poland; moreover, free-living amoebae have been isolated from natural water bodies including lakes, ponds, rivers, and lagoons of the West Pomeranian and Lubuskie area, in tap water of the water supply system of Szczecin city, in surface water layers, and in water with sediment in northern Poland at the area of the cities Gdańsk, Gdynia, and Sopot, as well as in swimming pools and fountains in western Poland [44–50].

At present, the approach based on genotype identification is more often applied to detect and characterize* Acanthamoeba* strains from environmental and clinical samples below the genus level. There is evidence that among seven genotypes detected in patients with AK about 90% of incidences are linked with the T4 genotype [3, 4, 17, 24]. In our investigations, all pathogenic strains of AK were identified as belonging to the T4 genotype. This is in accordance with the fact that this genotype is considered to be the most common cause of the vision-threatening eye disease. Our previous [16, 31] and this study are the only in that the molecular assessment was performed in diagnosed pathogenic* Acanthamoeba* isolates that originated from Polish patients with drug resistant* Acanthamoeba* keratitis. Complete results of these examinations will be reported in detail in a separate publication.

It is considered that the leading risk factor for* Acanthamoeba* keratitis is contact lens wear. After the first case of AK associated with contact lenses in Central Europe was reported from Germany, more incidences were recognized in different countries and an association between contact lens wear and* Acanthamoeba* keratitis was revealed [51, 52].

Nevertheless, occasionally* Acanthamoeba* corneal infections are also detected in persons not using contact lenses. A corneal epithelial injury, eye surgery, and, especially, an exposure of the eye to water or moist soil in which* Acanthamoeba* forms exist are considered as other important risk factors for acquiring AK [2–4, 17, 53].

Recently, the popularity of contact lens use is rising, and severe AK cases are reported with increasing frequency year after year, particularly in contact lens wearers (85% of all incidences), from various regions of the world, including Poland. Since the first incidences of AK were reported [54], further AK cases have been described in Poland [16, 31, 46, 47, 55].

All patients with keratitis symptoms from whom the corneal isolates were analyzed in our studies at the beginning were assessed to search for/confirm the etiological agent of the disease.

Lorenzo-Morales et al. [17] “the most important step in AK diagnosis is to think of it.” In many* Acanthamoeba* cases there is a history of misdiagnoses and improper therapy; also in the ten cases finally included in our study, some diagnostic difficulties and prolonged disease process occurred. The direct detection of etiological agents is considered as the only reliable diagnostic method for AK; simultaneously, cultivation remains the gold standard for* Acanthamoeba* laboratory diagnosis [17]. It is noteworthy that examiners have to be familiar with morphological characteristics of* Acanthamoeba* spp.

Currently, it has been shown that both environmental and clinical* Acanthamoeba* strains/isolates vary in their pathogenicity; they may be virulent, weakly virulent, or nonvirulent [4]. Simultaneously, the ability of* Acanthamoeba* to grow at high temperatures is considered to be correlated with the pathogenicity of* Acanthamoeba* isolates and to be a good indicator of the pathogenic potential of a given isolate. Thermal tolerance examinations and growth at high temperature are used as indirect markers of* Acanthamoeba* virulence/pathogenicity of some environmental samples [4, 17, 32].

Results of our earlier investigations [33] on environmental strains of* Acanthamoeba* in terms of their temperature tolerance showed that the amoebae may grow at 37°C, a temperature that is higher than that in which they had been cultured for many months; at the same time, the number of trophozoites of this strain was ~10% lower than at 26°C.

In general, our experimental investigations of ten pathogenic strains revealed the ability of all isolates deriving from AK cases to grow in higher temperature than 27°C at which they had been cultured for many months. The maintenance of metabolic activities was expressed in the relatively high population density that characterized all pathogenic* Acanthamoeba* isolates incubated in parallel at 37°C and 42°C.

It is noteworthy that cultures of the environmental Neff strain exhibited optimal growth at 20°C and weakest growth in temperatures higher than 27°C. Corneal pathogenic isolates developed well also at higher temperatures.

## 5. Conclusions

*Acanthamoeba* keratitis is a vision-threatening emerging eye disease caused by the facultative parasite* Acanthamoeba*, ubiquitous in human environments. The leading risk factor for the disease is contact lens wear, steadily rising in popularity; thus, AK is detected with increasing frequency worldwide, including Poland. Awareness of these risk factors and thus strict hygiene while cleaning and using contact lenses are crucial as preventive measures. The diagnostic and therapeutic difficulties, coupled with severe course of the disease in the cases analyzed in the current study, were due to unspecific clinical symptoms, misdiagnosis, and resulting delay in suitable treatment, as well as resistances to antimicrobial and antiparasitic therapy.

It was shown in our study that the ability of* in vitro* cultured corneal* Acanthamoeba* isolates to adapt to higher temperature was typical for all pathogenic isolates monitored. At the same time, the* in vitro* metabolic activities of these strains were also maintained at 20°C.

In our opinion, the pathogenic strains are not just thermotolerant but rather have a wide adaptive capability.

Our study is the first detailed study from Poland providing evidence of the significant role of adaptability to temperature changes as one of a complex of contributory factors allowing free-living amoebae to exist as parasites, namely, as the causative agents of AK, a serious human eye disease. Nevertheless, from which sources our AK patients acquired their infections remains uncertain, whether they came from natural or from man-made habitats. Also, which mechanisms enable particular strains to grow at a wide temperature range remains unclear, while others do not show this ability. These remaining areas need further studies if we are to understand the epidemiology of these opportunistic infections and to prevent future cases.

## Figures and Tables

**Table 1:** Compilation of data of *Acanthamoeba* T4 isolates of five severe AK cases with delayed proper diagnosis, resistant to chemicals applied.

*Acanthamoeba* strain	Probable risk factors	Microorganisms detected	The first-time *Acanthamoeba* detection	
			Stage	Material
I-1	Swimming in a lake	*Acanthamoeba* sp.	Moving trophozoites with acanthopodia	Corneal scrapings

I-12	Contact lens	*Acanthamoeba *sp.	Viable trophozoites, rounded forms	Corneal scrapings

I-13	Swimming pool, contact lens	*Acanthamoeba *sp. *Candida *sp.	Trophozoites, cysts	*In vitro* cultures

I-16	Contact lens	*Acanthamoeba* sp. *P. aeruginosa *	Trophozoites, cysts	*In vitro* cultures

I-19	Contact lens	*Acanthamoeba* sp. *E. faecalis*	Numerous cysts	Corneal scrapings

**Table 2:** Comparison of T4 strains from AK and *A. castellanii* Neff strain cultured *in vitro* at 20°C, 26°C, 37°C, and 42°C.

*Acanthamoeba* strain	Range of overall *Acanthamoeba* number (×10^2^)			
	Range of cysts (%)			
	20°C	27°C^*∗*^	37°C	42°C
I-1	21.1–23.3 0.8–1.5	62.2–138,2 0–0.8	30.0–51.1 0–1.2	17.8–38.2 0–3.5

I-13	20.1–34.4 0–1.7	120.0–207.7 0–1.0	46.6–78.9 0–1.3	12.2–26.7 0–3.0

I-16	25.3–26.1 0–1.5	123,3–164.4 0.9–1.0	52.2–74.4 0–0.8	23.2–29.0 0–3.0

I-19	32.7–34.0 0–0.7	85.5–145.5 0–0.2	44.0–102.2 0.5–0.9	22.0–27.8 0–1.1

Neff	378.1–424.6 0–0.3	102.0–176.7 0	3.3–7.8 0–4.2	2.2–8.2 0

The level of statistical significance was set at *p* < 0.05.

^*∗*^Data from exponential phase of population growth.
